# Nutritional Status of Orang Asli in Malaysia

**DOI:** 10.21315/mjms2022.29.3.3

**Published:** 2022-06-28

**Authors:** Janice Ee Fang Tay, Vaidehi Ulaganathan, Goh Yoke Lian Kua, Mulki Abdullahi Adan, Sook Yee Lim

**Affiliations:** Faculty of Applied Sciences, UCSI University, Kuala Lumpur, Malaysia

**Keywords:** nutritional status, dietary patterns, Indigenous Malaysian

## Abstract

The national government policies and Jabatan Kemajuan Orang Asli (JAKOA) have to put more effort to improve the quality of life for Orang Asli communities. Over the years, under the government-sanctioned relocation programme, many Orang Asli groups were moved to a more developed and urban area. They were given proper facility, healthcare service to ensure the improvement of overall well-being. While undernutrition among the Orang Asli remains a major health issue, evidence has shown that overweight and obesity in this population are increasing. This observation might be attributed to an urbanised lifestyle that often leads to unhealthy dietary patterns, leading to an increased prevalence of obesity, chronic diseases, food insecurity and unhealthy diet intake. The nutritional transition should be capture for a better understanding of Orang Asli nutritional status. This review assessed the nutritional status and its related key factors among *Orang Asli* population in Malaysia.

## Introduction

Indigenous people can be defined as a minority ethnic group of people sharing common characteristics and maintenance within the ancestral territories of cultural, social, economic and political entities (1). To date, indigenous people in Malaysia have compromised about 13.8% of the country’s total population of 31.7 million. The indigenous people are not viewed as a homogenous population group; they consist of various ethnolinguistic communities living in Peninsular Malaysia, Sarawak and Sabah in eastern Malaysia, practising their own language and culture. In Peninsular Malaysia, the indigenous population known as Orang Asli were classified into three sub-tribes, including Senoi, Proto-Malay and Negrito, each of which has several sub-ethnic groups. In Sarawak, 40% of the population was made up of Dayaks, including Iban and Bidayuh. On the other hand, Kadazan-Dusun, Bajau and Murut, with 60% of the state population, were the main indigenous groups in Sabah (2).

According to the Malaysia government, the Aboriginal Peoples Act 1954 (ACT 134) had implemented to recognise this community. Since 1957, government and non-governmental organisations (NGOs) have embarked on numerous development projects to promote the quality of life of the communities of Orang Asli, such as educational, financial, healthcare, resettlement, housing opportunities and many other initiatives. Despite the planned development, this population still remains socioeconomic marginalised and negative health aspects because they live in forest areas and still follow traditional lifestyles that are strongly influenced by the environment and cultural traditions of ancestral origin. This resulted in high mortality among the Orang Asli population that has limited access to modern medical facilities. The existence of a high prevalence of health problems like undernutrition, infectious diseases (e.g. measles and malaria) and chronic disease is a strong sign of significant shortcomings in Orang Asli social and public health policies. Other culprits resulted in a poor quality of life, including genetic vulnerability, socioeconomic disadvantages, resource alienation and political injustice (3–4). In addition, the process of urbanisation caused Orang Asli people exposed to market foods and other cultures, which can adversely affect their preference for food and lifestyle, leading to a rise in the risk of chronic diseases (5).

Recently, the Malaysian government had introduced the National Plan of Action for Nutrition of Malaysia III (NPANM III), 2016–2025, highlighted ‘the second Sustainable Development Goals (SDGs) that are zero hunger to end hunger, achieve food security and improve nutrition to tackle the high prevalence and effects of malnutrition collectively’ (1). However, the overall health status of the Orang Asli population is still far behind compared to other major ethnic groups (e.g. Malay, Chinese and Indians) and should not be neglected. This review was carried out to fill the information gap by identifying the key factors related to the nutritional status of the Orang Asli population.

### Nutritional Status

The nutritional status of Orang Asli is typically low, especially among women and children. Women’s nutrient intake is largely below the amount required. Research conducted in Pahang showed that less than half of the women had normal weight (6). Research in the Krau Wildlife Reserve, among adult Orang Asli from the Che Wong tribe showed that 13.8% of men and 25.0% of women were underweight (7). The prevalence of protein and vitamin deficiency was observed in more than a third of the surveyed population groups (8). Factors such as poverty, low diet consistency, inappropriate cultural values, lack of dietary awareness, poor hygiene practices and elevated helminthic infestations are due to the poor nutritional status of Orang Asli.

### Body Mass Index

Body mass index (BMI) is the most popular method for nutritional assessment whereby it captured the bodily composition in relation to weight and height status. According to World Health Organization (WHO) (9), BMI defined as an individual’s weight (kg) divided by the square of height (m) and classified into several ranges including underweight (< 18.5 kg/m^2^), normal weight (18.5 kg/m^2^–24.9 kg/m^2^), overweight (25.0 kg/m^2^–29.9 kg/m^2^) and obese (≥ 30.0 kg/m^2^). BMI greater than or equal to 30.0 kg/m^2^ is considered to be caused of abnormal or excessive adiposity. Five studies were included in this review for analysis (7, 10–13). Yusof’s (10) study (*n* = 138) showed that the mean BMI of Orang Asli adults in Lembah Belum, Gerik in Perak state were reported to be 20.8 ± 4.1 kg/m^2^, with mean BMI values for male and female as 20.5 ± 2.9 kg/m^2^ and 21.0 ± 4.77 kg/m^2^, respectively, and there was no significant difference reported between male and female (10). There were 26.7% of the respondents could be considered malnourished, and 10.1% of them were either overweight or obese (10). Another study (*n* = 57) has reported that the mean BMI was 21.83 ± 3.4 kg/m^2^ for men and 21.31 ± 4.05 kg/m^2^ for women from Che Wong tribe in the Krau Wildlife Reserve in Pahang State. Among the male respondents, 13.8%, 72.4% and 10.3% of them were underweight, normal and overweight, respectively, while for female, 25.0%, 46.4% and 28.6% of them were underweight, normal and overweight, respectively (7).

Based on Azuwani’s (11) study (*n* = 138), the data showed that the mean BMI was 25.7 ± 4.61 kg/m^2^ for Orang Asli in Cameron Highlands. According to the BMI categorisation, most of them were normal/healthy (37.7%), followed by obese (34.8%), overweight (25.4%) and underweight (2.2%). Another study (*n* = 351) by Chang (12) reported that the mean BMI for Sarawak Orang Asli was 30.5 ± 4.5 kg/m^2^. Specifically, female respondents (30.9 ± 4.80 kg/m^2^) had significantly higher mean BMI than males (29.1 ± 3.08 kg/m^2^). More than 40% of the respondents were obese and 54.7% were overweight (12). Another study (*n* = 58) conducted in 2018 showed that the mean BMI of respondents from Temiar Orang Asli Community in Kuala Betis, Gua Musang in Kelantan State was 25.2 ± 5.3 kg/m^2^ where there was no significant difference between men and women. According to the WHO (9) classifications, 9% of respondents were listed as underweight, while 40% of them were normal and 51% of them were overweight or obese.

Despite the inconsistent findings, the prevalence of underweight and overweight in the studies reflecting the existence of double burden of malnutrition in the Orang Asli communities. This phenomenon might be due to the rapid transition in relation to the demographic, socioeconomic, traditional dietary patterns, and lifestyle, may be experiencing nutrition transition (14). This rapid change from a highly mobile lifestyle and traditional ethics diet to westernised diet, increased consumption of animal-source food, high sugar, salt and fat, greater calories intake, limited fibre intake, and sedentary lifestyle could be the reasons contributing to overweight and obese. Besides, the changing patterns in food consumption to monotonous diets with low nutrient adequacy could lead to micronutrients deficiencies and underweight, especially a concern among Orang Asli. Malnutrition is well known to be associated with both short- and long-term harmful consequences on the development of chronic diseases, which will in turns dramatically increase the risk of mortality and morbidity as well as impaired the social activity, economic productivity, and psychological health (14). The high prevalence of malnutrition among Orang Asli could be due to the occurrence of nutrition transition, however, limited strategies to overcome this and further research is required to identify better strategies to address the nutrition transition-related consequences in Orang Asli communities.

### Body Fat Percentage

The proportion of body fat is the total body fat mass divided by total body mass and multiplied by 100, indicating an individual’s body composition (15). However, BMI is the measurement of the ratio between body weight and body height, it cannot reveal the fat distribution in the body. Therefore, it is necessary to include both BMI and body fat percentage (BFP) to have an accurate diagnosis during a health check procedure. WHO (16) has proposed a definition of obesity in males as greater than 25% of body fat and in females as greater than 35% of body fat. Similar to BMI, BFP could reflect the level of adiposity and providing a better overview of individual nutritional status in terms of the associated obesity-related diseases.

There are three studies (10–11, 13) involved in the body fat analysis for Malaysia Orang Asli. The mean of body fat was 18.3 ± 6.06% (15.9 ± 5.78% for male and 19.8 ± 5.77% for female). According to WHO (9) classification, 53.4% (body fat < 18%) respondents were lean, 45.8% (18 ± 32%) were normal and only 0.8% were obese (body fat > 33%). Azuwani’s (11) research study showed that the mean of the body fat for Orang Asli in Cameron Highlands were 25.3 ± 8.8%. Most of the participants had an acceptable percentage of body fat (36.2%), followed by obesity (31.2%) and fitness (16.7%) (11). The results from the third study (*n* = 58) show that 2% of respondents had low levels of body fat, 29% of them had normal (mean = 24.52 ± 4.65%) and 69% of them had a high (mean = 28.65 ± 4.99%) and very high (mean = 37.37 ± 6.87%) body fat. Overall, these findings indicate that most respondents were deemed overweight or obese (13).

Two of the results (10–11) have shown that most of the respondents have acceptable body fat, while one of the studies (13) shows that most respondents have high body fat and could be due to high carbohydrate intake (60.72 ± 32.15%) that is slightly exceeding the carbohydrate intake of the general Malaysian population of 46%–60%. They rely on food rich in carbohydrates, such as tapioca, yam and wheat flour. However, more studies are required to indicate and provide more information on the body fat of Orang Asli Malaysia.

### Waist Circumference

Apart from BMI and BFP, waist circumference (WC) is also an important health indicator that helps to screen for potential health risks associated with overweight and obesity. WC is measured at the end of several consecutive natural breaths, the midpoint between the top of the iliac crest and the lower margin of the last palpable rib in the midaxillary line is determined at the level parallel to the floor (17). The data were analysed using cut-offs points where for Asians are 90 cm for men and 80 cm for women (18). WC is one of the most accurate anthropometry methods to indicate abdominal fat (17).

There are four studies that have examined the WC of Orang Asli (7, 9, 11, 13) in Malaysia. The first study has shown that the mean of WC was 71.1 ± 7.50 cm (72.5 ± 8.26 cm for male and 70.2 ± 6.86 cm for female), with only 0.8% of them exhibited central obesity (> 108 cm, for male and > 88 cm for female) (10). Another study (*n* = 57) conducted in 2010 indicated that the mean WC was 74.4 ± 6.09 cm and 73.92 ± 6.80 cm for males and females, respectively (7). While none of the men had a WC at risk, 21.4% of the women had a WC > 80 cm at risk (7). The study published by Azuwani (11) (*n* = 138) showed that the mean of WC for respondents were 79.2 ± 12.53 cm, where 26.8% of the respondents had central obesity according to the waist-hip ratio, while 13% of them had central obesity according to the WC. Another study (*n* = 58) indicated that the mean of the normal WC was 73.46 ± 11.27 and 96.51 ± 7.20 for the at-risk category, where 16% of respondents exhibited central obesity (13). From the results, it can be summarised that most of the Orang Asli has a normal WC, with minimal percentages of them were reported to have a centre obesity problem.

### Food Insecurity

According to the Food and Agriculture Organisation of the United Nations, food security can be defined as a condition in which all people have access to adequate, nutritious and safe food economically and physically at all times to meet the nutritional needs and tastes of active and healthy life (4). Collectively, food security issues are identified among the Orang Asli population of Malaysia. A total of three studies had reported on the food insecurity status among Orang Asli (1, 19–20). The food insecurity severity level can be divided into four categories: i) food secure; ii) household food insecure; iii) individual food insecurity and iv) child hunger (21). Zalilah and Tham (19) assessed the prevalence of food security among pre-schoolers (3–6 years old of age children) of Temuan households (*n* = 64). A total of 82.8% of the households reported some household food insecurity with child hunger (28.1%), individual food insecurity (32.8%) and household food insecurity (20.3%) (19). The study indicated that subjects with child hunger could experience household food insecurity and individual food insecurity (19). In another study conducted by Nurfahilin and Norhasmah (20) on female Orang Asli (*n* = 92) from Gombak in Selangor State, about 88% of the household were food insecure, with 48.9% of them reporting household food insecure, 21.7% individual food insecure and 17.4% child hunger. In a study on Mah Meri women (*n* = 222), 82.9% of households reported that they experienced food insecurity, with 29.3% subjects experiencing household food insecure, followed by 23.4% subjects experiencing individual food security, and lastly, 30.2% of the subjects were in the child hunger category that experienced the most severe level of food insecurity (1).

### Food Insecurity and Nutritional Status

Food insecurity is closely related to poor nutritional status, including malnutrition and malnourishment, which can be defined as experiencing a poor or inadequate diet that leads to malnutrition. Five research were included in this topic (1–2, 4, 19–20). Khor and Shariff (2) highlighted that Orang Asli’s poor health and nutritional status might be largely due to food and nutrition security issues, especially affecting women and children. Childhood undernutrition, including underweight and stunting, are commonly observed, while overweight and obese are high prevalence among adults. Insufficient food and nutrition supply that were unhealthy and imbalanced may result in various health issues such as anaemia, lack of iodine, vitamin A deficiency and mumps, and substance abuse among Orang Asli communities (4).

Based on a study on Mah Meri women (*n* = 222), household food insecurity (28.5 ± 5.8) was significantly correlated with a higher mean BMI than individual food insecure (26.7 ± 7.3) and child hunger (25.8 ± 4.6) after controlling for age (*P* < 0.025) (1). This indicated that women at household food insecure appeared to be overweight or obese. This observation may be explained as a cyclical phenomenon as access to food is limited during the time of food insecurity, and the household may face food shortages. Therefore, when food supply is adequate, the perceived anxiety or depression about future food scarcity causes an individual to have binge eating. In order to sustain their quantity of food consumption, household food insecure women may opt for less costly and high calorie-dense food, which contributes to an increase in energy intake, thereby gaining weight. In contrast, research conducted on Orang Asli women in Gombak, Selangor (*n* = 92) stated that the BMI and WC were found no statistically significant difference between the food secure and food-insecure groups (20). This study also showed that more than half of the respondents were reported as obese and overweight, 63.7% among the food secure group and 59.3% among the food-insecure group. For women with household food insecurity (27.54 kg/m^2^) and individual food insecurity (27.82 kg/m^2^), the mean BMI was slightly higher relative to child hunger (24.85 kg/m^2^). Among all, 2.2% of household food insecurity and 25% of child hunger were found experiencing underweight, whereas, in terms of at-risk WC, more women under the food secure category (72.7%) were a higher risk compared with women in household food insecure (66.7%), individual food insecurity (60%) and child hunger (50%) (20).

Zalilah and Tham (19) assessed the dietary intake and anthropometric measurements of the pre-schoolers as proxies of hunger and malnutrition (*n* = 64). The food secure group had higher percentages of normal weight-for-age and height-for-age children compared to the food insecurity group. Among children in household food insecure, 46.2% of them were significantly underweight, 30.8% were significantly stunted and 7.7% were significantly wasted (19). For children in individual insecure groups, 47.6%, 57.2%, and 9.5% were found for significantly underweight, stunting, and wasting, respectively (19). Food and nutrition insecurity in these communities is partly responsible for the change in Orang Asli’s food systems, which could be attributable to the difficulties of preserving traditional food systems and increasing the availability, accessibility and acceptance of westernised food. Increased intake of cheaper energy-dense and nutrient-poor foods is likely to predispose children to poor growth and development due to limited financial resources and provide adults with excess calories that contribute to obesity and the associated implications of chronic metabolic diseases (2). Among Orang Asli children, the main indication of prenatal and postnatal undernutrition includes short maternal stature, low birth weight, prematurity, low dietary diversity, parasitic infections, insufficient sanitation and hygiene (4). In summary, household food insecurity was the most prevalent type of food insecurity reported, with a greater prevalence of females with higher BMI and malnourished children.

## Food Insecurity and Dietary Intake

### Dietary Energy, Macronutrients and Micronutrients

Three studies were included in investigating the relationship between food insecurity and energy and nutrient intakes (1, 19–20). Among the Temuan pre-schoolers (*n* = 64), the study had reported that intakes of macronutrients and micronutrients seemed to be decreased as food security worsened (19). The mean percentage of recommended dietary allowance (RDA) for calories among household food insecure is 78.7%, followed by 53.9% at individual insecure level and then 61.1% at child hunger. The RDA percentage of protein intake was found to be a similar pattern as calories intake with 177.6% (household food insecure), 144.8% (individual insecure) and 123.2% (child hunger). For micronutrients intake, the calcium and iron intakes were found to be less than 2/3 of RDA for individuals insecure (calcium - 54.7%, iron - 46.4%) and child hunger (calcium - 52.7%, iron - 49.8%). On the contrary, the average intake of vitamin A (112.2%–165.0% RDA) and vitamin C (206.3%–269.3% RDA) were reported to be high across the food insecurity group. Nevertheless, the actual intake of vitamin C may be lower than the reported values as the values included in the Malaysian food database did not take into account the losses (40%–70%) incurred during food preparation and cooking. Thus, the real intake could be fall between 70% and 140% RDA instead of the high amount as reported (19).

Another study (*n* = 92) was 1087.6 ± 692.7 kcal, 877.0 ± 323.5 kcal and 955.4 ± 409.0 kcal, respectively, on the differences in women’s energy and nutrients intakes by food security status, mean calorie intakes among respondents in household food insecurity, individual food insecurity, and child hunger (20). In household food insecurity, the mean carbohydrate, protein and fat were found to be the highest at 150.2 ± 118.9 g, 48.9 ± 28.5 g and 31.0 ± 24.6 g, respectively. Meanwhile, the mean consumption of vitamin C was higher for food insecurity in households (47.8 ± 55.2 mg) and lowered for the food secure group (12.9 ± 14.4 mg). However, there was not statistically significant mean difference in the intakes of energy, protein, carbohydrate, fat, vitamin C, iron and calcium. Vitamin A was the only nutrients found to have a significant difference between the group that highest in household food insecurity (232.0 ± 561.0 mg retinol equivalents [RE]) and lowest in individual food insecurity (32.5 ± 70.8 mg RE) (20). The high intake of protein observed was due to a combination of plant and animal proteins, including fish and eggs that were the main protein sources, whereas high consumption of green leafy vegetables that are highly nutritious in vitamins A and C contents contributes to the high vitamins C and A (19). Research conducted by Pei, Appannah and Sulaiman (1) assessed the consumption level of fat and sodium intake using the Malaysian healthy eating index (HEI) (*n* = 222). The research indicated the women from the child hunger group were significantly correlated with higher mean sodium (*P* < 0.001) and fat (*P* < 0.05) nutrient score compared with other groups. A higher Malaysian HEI score indicating lower consumption of sodium and fat. Women from child hunger showed to experience severe food insecurity, causing them to reduce the frequency of meal intake and daily expenditure on basic food to provide their children’s needs, thus resulting in low consumption of sodium and fat. However, during food insecurity, consumption of available food in the household such as fried rice, soy sauce, and salted fish anchovies resulted in the higher intake of fat and sodium observed among women from household food insecure (1). In short, the intake of macronutrients and micronutrients are generally worsened across the food insecurity groups, given that individuals from individual insecure and child hunger groups were the most at risk to poor dietary intake despite the intake of the selected nutrients such as vitamin C, vitamin A and protein is higher than the RDA. This unbalanced nutrition can still contribute to considerable adverse effects on health.

### Food Groups

Two papers were studied on the food security status association with consumption of different food groups (1, 16). The recent study (*n* = 222) indicated that respondents of the individual food-insecure and child-hunger groups had lower Malaysia HEI scores; specifically, for grains and cereals with a mean score of 8.0 ± 1.7% comparing with 8.9 ± 1.4% in the secure group as well as meat, poultry and egg consumption with a mean score of 4.0 ± 3.3% comparing with 6.0 ± 3.6% in the secure group (1). The study (*n* = 64) conducted by Zalilah and Tham (19) that further analyses of food groups were done to facilitate the explanation for nutrients intakes using RDA. Among the five food groups, the highest total mean scores of the food group were observed in the cereals, cereal products and tubers group (1.48 ± 0.71), indicating more individuals have met these groups’ dietary goal. However, fruits and milk and dairy products scored lower total mean scores of food groups, 0.28 ± 0.60 and 0.22 ± 0.55, respectively (19). Low consumption of milk and dairy products may be due to the period that children had stopped drinking milk after weaning as well as the low level of acceptability of dairy products. Although fish was reported to have higher total mean scores (0.95 ± 0.72), the number of servings may not contribute relevant nutrients to the diet (19). Such low food group intakes may explain the low calorie, calcium and iron intakes among the children because these food groups provide most of the nutrients in the diet that essential in contributing to childhood growth and health. Additionally, the low consumption of fruits among the Orang Asli population could be due to the less availability of seasonal fruits around their settlement (1).

### Diet Quality and Patterns

Two papers documented data on the relationship between food insecurity and diet quality (1, 19). Zalilah and Tham (19) assessed the diet quality index using the recommended serving size on the Malaysian Food Guide Pyramid for children (19). Findings suggested that the diet quality of pre-schoolers (*n* = 64) decreased as household food insecurity worsened. The prevalence of children with poor diet quality index from the individual insecure group (81.0%) is higher than that of the household food insecure (53.8%). The low availability of food supply for consumption are the key factors that restrict the food variety and hence the quality of diet (19). A study on Mah Meri women (*n* = 222) indicated a strong correlation between food security status and Malaysian HEI (1). The analysis indicated that the women from individual food insecure (45.3 ± 7.3%) and child hunger (44.4 ± 6.3%) groups were significantly correlated with a lower mean Malaysian HEI score than the food-secure category (48.4 ± 7.3%) after controlling for age. The diet quality increased as the mean Malaysian HEI score increased. The food insecurity associated with low diet quality could be due to lower intake of components such as fruits, milk and dairy products, meat and poultry. Besides, the limited economic access to a variety of food options or the increasing food prices that restrict the ability to provide sufficient food has resulted in a general decrease in food intake and diet quality (1). Other potential tools that can be used to assess the diet quality among Orang Asli population, including Healthy Diet Indicator, Nutrient-Rich Food Index and Easting Choices Index. Nutrients-Rich Food Index assesses diet quality by rating the nutritional value of the food. The eating choice Index is utilised to distinguished between healthy and unhealthy eating choices by referring to the dietary recommendations (22–23). As the food security worsened, the overall diet quality deteriorated, posing increasing nutritional threats to Orang Asli health.

## Risk Factors of Food Insecurity

### Socioeconomic and Sociodemographic

Table 1 shows the risk factors associated with food insecurity among Orang Asli. Socioeconomic and demographic had been studied and proved to be the determinant of food security. A total of four papers evaluated this relationship (1, 4, 19–20). Pei, Appannah and Sulaiman (1) (*n* = 222) suggested that as the household income, per capita income and food expenditure decreased (*P* < 0.001), the food insecurity worsened significantly. Women from the child hunger group were shown to have a significantly higher number of children (3.1 ± 1.7) and a larger household size (5.2 ± 1.7) than that in the food-secure group (number of children: 2.6 ± 2.1 and household size: 4.6 ± 2.6) (1). Another study (*n* = 92) suggested that difference in school years, number of children, monthly income and household size were identified to have significant difference between food security status (20). The findings indicated that the mean score of schooling for group child hunger (4.31 ± 2.11) was significantly lower than that from group household food insecure (7.6 ± 4.12). A higher mean score number of children were found in child hunger (4.44 ± 2.28) and less in food secure (2.27 ± 1.74) and household food insecurity (2.31 ± 1.90). Whereas, the average household size score for child hunger (8.00 ± 3.18) was significantly different from the food-secure group score (4.00 ± 1.90). In food secure group (RM1,988.27 ± RM1,073.50), the average monthly total income was higher and lower in the severe child hunger group (RM1,051 ± RM761.04) (20). A study (*n* = 64) on the prevalence of household food security among the Temuan community, based on household income and per capita income, improved household food security as household income and per capita income increased (19). The findings indicated 17.1% of the group secure and 32.2% of household insecure having income less than RM500 (19). A low socioeconomic status attributes, including low monthly income, low per capita income, low education status and lack of regular employment among household, can cause food insecurity (4).

The decline in household income across the food security status may be related to income instability from the culture-dependent job, in which bad weather often affects fishing and agricultural crops. Low household income indicates lower purchasing power, not allowing them to obtain sufficient food for household or to buy a range of foods that could be contributed to a balanced diet that is essential for growth and health, even at high prices (1). However, a recent study stated that income as an indicator for the risk of food insecurity might not be the most suitable indicator (19). This is due to the less consideration in the differences of other expenses within the household, and the annual income-based poverty is not prone to sudden economic shift that may lead to temporary household food insecurity outbursts. It has been proposed that the impact of recent economic shifts on household finances such as unexpected costs, work reductions or the addition of a new member of the household in recent months be considered when examining the relationship between deprivation and food insecurity. Furthermore, an increase in the number of children will lead to an increase in the expenditure on child education and healthcare as well as general household expenses. Restricted financial resources in a greater household size may continue to experience larger uncertainty due to food insufficient as the need for food increases linearly with the number of household members, thus resulting in a higher risk of food insecurity for the family (1, 4, 19). To come to the point, the existing link between socioeconomic and demographic factors with the food insecurity should be investigated further to determine the extent to which economic interventions and social supports are crucial in improving the food security and nutrition of Orang Asli.

### Food Source

The difficulties and obstacles in obtaining food sources as risk factors for the Orang Asli population to experience food insecurity were reported in one study. Gan et al. (5) conducted a qualitative study among Jahai sub-tribe communities on food acquisition and barriers in obtaining traditional and market foods (*n* = 28). The study provides evidence on the barriers to obtain an adequate amount of traditional and modern food (5). Food shortages were primarily due to the deterioration of the natural climate that affected the reproduction and survival of traditional food species, and the centralised resettlement forced them to move to other places. The low purchasing power was mainly reported under the restriction of modern practices. In this analysis, low purchasing power was attributed to low socioeconomic status, such as high participation in self-employed occupations and low educational qualifications among the informers. Besides that, high transportation cost was also reported as barriers to secure adequate food supply for the households (5). While travelling to nearby towns for food procurement already incurs a significant cost of fuel, along with minimal income, strained the Jahai’s financial position and reduced its purchasing power even further (5).

One of the food-related activities of Jahai sub-tribe is food sharing which is required to boost the strong relationship between family members and neighbours. However, this tradition is reported as one of the barriers as, under certain circumstances of food insecurity, sharing of food could be a burden to the household. High demand for food was identified as obstacles in securing sufficient food for a household in this study. Typically recorded high demand from those of large household size, they expected to obtain less and poorer quality food in the worst situation compared to those of smaller household size. Wild animals were treated as challenges faced during traditional food-seeking practices when insufficient food supply from agricultural activities (5). Food insecurity not only linked with the economic resources, the environmental, transportation and cultural factors can create complex challenges to food sources and subsequently raise their vulnerability to food insecurity and poor nutritional outcomes. Building a supportive environment for enhanced food security is therefore important to ensure the capability for Orang Asli to access to enough nutritious and safe food.

## Coping Strategies

Many studies have identified coping mechanisms for dealing with temporary food insufficiency, also known to reduce the uncertainty of household insecurity. Three of the studies reviewed and reported the coping strategies adopted during the food insecurity period (4–5, 20). According to Nurfahilin and Norhasmah (20) (*n* = 92), coping strategies were classified into three categories which are food-related coping strategy, non-food related strategy and other related coping strategies. Among food-related strategies, most of the women adopted the coping strategies of consuming whatever food is available around the house (66.3%) and using less expensive food (64.1%). For non-food-related coping strategies, many of them adopted the strategy of being thrifty in using money (82.6%) and planning for expenses (82.6%). They also implemented other related coping strategies, such as catching or fishing fish from rivers (22.8%) and depending on forest sources for food (26.1%), which are known as the traditional seeking food practices (20). A qualitative study (*n* = 61) was performed to assess the types of coping strategies adopted and the associated severity level for food insecurity among women from three ethnic groups (Senoi, Proto-Malay and Negrito) during the period of food insecurity. A total of 29 strategies covering both traditional food searching and modern economy practices were identified and further categorised into two themes: food consumption and financial management coping strategies. Among the food consumption coping strategies, ‘searching for food from food for surroundings’ (95.1%) under the sub-themes diversification of food sources was the most common food consumption coping strategy, followed by request or borrow food (86.9%) under the same sub-themes, and then eat the less preferred meal (83.6%) under the sub-theme dietary change. Less preferred food consumption represented an inability to be satisfied with the food, particularly where it was a food that had been persistently eaten and that the participants would become bored with eating. Besides, decrease in the number of people (visit friends or relatives during a meal - 21.3% and ordering children to eat at homes of neighbours or relatives - 11.5%) and rationing (skipping meals - 77.0% and reducing portion size - 75.4%) are also adopted as food consumption coping strategies (4).

The financial management coping strategies comprised three sub-themes, including increase household income, reducing the expenses involved for children of school age and reducing the expenses for daily necessities (4). Seldom bought clothes (45.9%) was reported to be the most practised financial management coping strategy, followed by getting involved in odd jobs (44.3%). Seldom buying clothes had shown varying degrees of money-saving behaviour, so that more money can be devoted to food in any situation (4). Regarding the severity of coping strategies, the 29 identified coping strategies were categorised into less severe, severe and very severe based on the degree of household food insecurity when they adopted the following coping strategies (4). Eight coping strategies were grouped under the less severe category. Eating less preferred food and find food from surroundings were considered to have equal severity as less severe (4). A total of 17 coping strategies were labelled as severe, namely borrowing money to purchase food and skip meals. Only four coping strategies were categorised under a very severe group, for instance, stay hunger and stop schooling for children. The identified severity level for each recognised coping strategy had provided evidence to show that food security is a managed process (4). Gan et al. (5) reported that six techniques were practised by hardcore poor households of Jahai sub-tribe to obtain food for their family (*n* = 28). Techniques including buying groceries from local grocery shops or food outlets, seeking edible plants (e.g. fern shoot and tubers) in the jungle, fishing at nearby lakes and rivers and hunting for animals in the forest were identified. As Jahai lives along the rivers and thus, fish is their primary food source. Besides, some of the Jahai households also receiving support from NGOs and engage in farming activities to sustain the household food sources (5). The ability to obtain food *via* traditional and modern means has not protected the Orang Asli from household food insecurity (5). In short, while coping strategies and techniques as social responses might temporarily assist Orang Asli to mitigate the negative impacts of food insecurity, long term financial and food compromise coping strategies can still bring health risks. Special attention should be paid to widow Orang Asli, with a focus on establishing a food security initiatives aimed at boosting social and financial protection schemes and enhancing the food security and nutrition of Orang Asli.

### Food Habits and Lifestyle

The food habits and lifestyle practised by the individual are crucial among the Orang Asli population as there is a link between health, nutrition and lifestyle. Adequate and healthy nutrition practices may reduce the risk of becoming sick and less vulnerable to diseases. However, modernisation and urbanisation have brought about a change in Orang Asli food habits and lifestyles that lead to the new age of disease (4). A total of three papers reported on the food habits and lifestyle practised by Orang Asli (3, 5, 24). A qualitative approach was conducted to determine the food taboos and avoidance practices related to lifestyle changes during pregnancy in modern times among Temiar women. The findings (*n* = 38) showed a total of 17 food restrictions were reported by participants and were separated into four groups: i) aquatic animal-fish; ii) animals; iii) plants and iv) processed foods. These food restrictions imposed on women during pregnancy are believed to maintain harmony with natural and spiritual forces and prevent any misfortune or calamity. Fear of complications during labour and childbirth, *sawan* or convulsions affecting the baby (such as foetal malformation) and twin pregnancy were the key reasons causing the practice of these food taboos. These food taboos practised might lead to an insufficient and imbalanced diet. Most of the traditional avoidance and dietary restrictions have been passed on to Temiar communities for generation and are still prevalent (24). Another study (*n* = 28) was conducted to establish an understanding of food preparation techniques practised by Jahai sub-tribe. The main cooking methods, including roasting and grilling, frying, simmering and boiling. Cooking oil and water have been the principal cooking medium among Jahai subtribe. Understanding the frequent use of such food preparation methods is important because methods of food preparation have been shown to affect the nutritional quality of food. Nonetheless, the effect of food preparation methods and the food taboos on the nutritional status of the Orang Asli population are important subjects to be explored in future studies (5). Aziz et al. (3), in their study (*n* = 191), reported that the lifestyle changes which unhealthy food habits and low physical activities adopted were observed among Orang Asli due to modernisation and urbanisation. In summary, unhealthy food habits in relation to food taboos, traditional food cooking methods and sedentary lifestyle are affecting the nutritional status among Orang Asli.

### Health Implications

#### Infection disease

Infection disease, especially intestinal parasitic infections, is the most prevalent among health implications correlated with clinical manifestations of malnutrition (e.g. protein-energy malnutrition) and deficiency of micronutrients. Among Malaysian Orang Asli, intestinal parasite infections remain a major public health issue where poor environment and sanitation, poor hygiene, unhealthy food habits, overcrowding, low educational achievement and deprivation are prevalent. There are two studies involved in the investigation of these infectious diseases among the Orang Asli population (25–26). The study by Al-Mekhlafi et al. (25) on the prevalence of *Giardia duodenalis* among aboriginal primary school children and its adverse effects on child growth in Pahang (*n* = 374) showed that a total of 22.2% of children were found to be positive for *Giardia* infection. There was a high prevalence of Giardia infection in children < 10 years old of age compared to those > 10 years old of age (27.4% versus 16.0%), but there was no statistically significant difference (*P* = 0.141). When compared between males (24.6%) and females (20.0%), there is also no statistically significant difference being found. With regards to the nutritional status, the mean weight and height of children among those infected with *Giardia* were shown to be lower. The mean weight (22.1 kg) of the participants infected with *Giardia* was lower than the mean weight of the non-infected participants (24.4 kg). Even though the mean height of the participants infected with *Giardia* (124.9 cm) was lower than the mean weight of the non-infected participants (127.1 cm), there was a statistically significant difference. Therefore, it is suspected that parasite infections contribute to child malnutrition through the progressive reduction of chronic inflammation and nutrient deficiency in digestion and absorption. Acute diarrhoea, malabsorption of fat, vitamins and D-xylose, and lactose intolerance are stated to cause *Giardia* (25). Another study (*n* = 341) among Iban tribe communities showed that the overall prevalence of parasitic intestinal infections was 57.5%, generally varied from 33.3% to 100% in all age groups with soil-transmitted helminths infections (50.4%) and protozoan infections (10%). Young children (55.3%) had a higher overall prevalence of infections compared to adults (6.7%). This may be because children were not aware of personal hygiene and good cleanliness practices or were unaware of the possibility of exposure to pathogenic organisms (26). The burden of infectious diseases among Orang Asli, in which children are the most susceptible group, can cause morbidity by compromising nutritional status.

#### Chronic diseases

Increased risk of chronic diseases such as cardiovascular diseases and diabetes may be detrimental to an individual’s life. It is important to investigate the factors associated with chronic diseases among Orang Asli population, and a total of two papers were included (3, 27). Researchers have assessed biochemical analyses to evaluate the impact of resettlement across six different sub-tribes (Inland: Bateq, Lanoh, Semai; Periphery: Kensiu, Che Wong and Kanaq) on changes in lifestyle and health status (*n* = 191). Participants from the Lanoh community showed a higher risk of diabetes, which could be due to insulin resistance, compared with other sub-tribes as the high mean level of insulin (176.95 pmol/L) hs-CRP levels (28.02 nmol/L) detected. By using Framingham Online Calculator, the cardiovascular diseases risk score was determined, where 42% of the male in inland have high Framingham risk score (FRS) (21%) and moderate (21%) FRS as compared to males in the periphery (16.7% FRS for each). A high-risk score (4.2%) of FRS was only found in inland among females. The changes in lifestyle and diet due to modernisation and urbanisation have also resulted in the consumption of fast food either inland or periphery among the Orang Asli population, which led to an increased risk of chronic diseases (3).

A cross-sectional study (*n* = 341) conducted among the Iban tribe population in Sarawak demonstrated the prevalence of anaemia by assessing the haemoglobin level, given 36.4% of the population with a mean Hb level of 126.0 g/L. The findings also indicated that females (39.8%) are more susceptible to anaemia compared with males (32.9%). The prevalence of anaemia was age-dependent, with a high prevalence recorded for adults 18 years of age and older (55.2%), followed by school children (25.6%) and young children (25.6%). Due to their physiological changes and menstrual blood loss, particularly during reproductive age, females are more likely to be anaemic. This research also illustrated that anaemia was significantly associated with parasitic intestinal infection (*P* < 0.001). It may be due to the synergistic influence of the species causing blood loss due to hookworm and reduced reabsorption or iron indigestion (26). Chronic disease is a global health concern, also seems to be an increasing public health issue in Orang Asli populations.

#### Metabolic impairment

Metabolic impairment, also known as a metabolic disorder, refers to the abnormal chemical reaction in the body that interferes with the process of metabolism, which causes the body to have either excess or insufficient quantities of essential substances required to remain healthy. One study that is linked to metabolic impairment among the Orang Asli population was found. Aziz et al. (3) conducted medical examination and biochemical analysis among Orang Asli in Peninsular Malaysia (*n* = 191). Males (inland and peripheral; 4.32 ± 1.03 mmol/L and 4.64 ± 1.03 mmol/L, respectively) showed better regulation of total cholesterol levels than females (inland and peripheral; 4.53 ± 0.95 mmol/L and 4.93 ± 1.01 mmol/L, respectively) concerning lipid profiles. Compared to women who stay at home and live a sedentary life, this tendency can be related to a higher level of male physical activity because they will have to earn or search for family food. Besides, the high percentage of presence of hyperinsulinemia and high hs-CRP levels were reported among all the participants. High insulin levels (> 173 nmol/L) were found in male inland (Semai - 16%; Bateq - 26%; Lanog - 20%) and periphery (Kensiu - 12%; Che Wong - 44%). Among the inlanders, female Bateq (50%) and Lanoh (54.55%) were reported to have a higher percentage of undergoes high hs-CRP levels (> 28.6 nmol/L) compared to other tribes. High levels of hs-CRP could be due to obesity that resulted from low physical activity and high intake of fatty foods or high calories meal, as well as a condition like injuries, illness and infections were prone to increase the level of hs-CRP and lead to determine the estimated risks falsely (3). Hyperinsulinemia and high hs-CRP levels are associated with insulin resistance and depressed cardiovascular antinomic function. These metabolic impairments are preventable and treatable by proper nutrition management and nutrition education to establish healthy lifestyle and diet, thus create a healthier Orang Asli communities.

## Conclusion

Double burden of malnutrition is recognised as a public health issues among Orang Asli communities, where modifiable factors including food insecurity, coping strategies, food habits and lifestyle have been demonstrated to impact the nutritional status of the Orang Asli in Malaysia. Food security and its associated risk factors play the major role in securing the nutritional status among Orang Asli communities, in which the initiated changes in lifestyle and dietary behaviour is likely to predispose Orang Asli to have greater exposure to chronic diseases. The findings not only warrant the attention of researchers to explore the underlying risk factor and potential consequences of the co-existence of malnutrition in the aspects of cultural, social, economic and environmental in Orang Asli communities, but also urge for the needs for immediate actions. Comprehensive public health policies and initiatives involving stakeholders at multi-level aiming at nutritional improvement, food environment in terms of accessibility and affordability to quality diet, and nutritional awareness along with socioeconomic and educational empowerment, may potentially to mitigate the issues. Additional efforts should be undertaken to ensure the effectiveness of these policy actions, as well as to consider the sociocultural sensitivity, to simultaneously relieve the Orang Asli malnutrition problem.

## Figures and Tables

**Table 1:** Risk factors associated with food insecurity among Orang Asli

		References
Demographic and socioeconomic characteristics		
Household monthly income Per capita income	Poor income stability from culture-dependent job such as fishing and agricultural activities Lower purchasing power	(1), (4), (16), (17)
Number of children Schooling children	Shift expenditure towards children education and healthcare	(1), (17)
Household size	Increased in general household expenses and food expenditure, experiencing food insufficient	(1), (17)
Food source (barriers)		
Food shortages	Deterioration of natural climate that affected the reproduction and survival of traditional food species	(5)
Transportation	High transportation cost to travel to nearby towns to purchase food	(5)
Food sharing tradition	Posing greater burden to household food insecurity	(5)
